# Acute Kidney Injury in the Patient with Cancer

**DOI:** 10.3390/diagnostics11040611

**Published:** 2021-03-29

**Authors:** Alejandro Meraz-Munoz, Amit Langote, Kenar D. Jhaveri, Hassane Izzedine, Prakash Gudsoorkar

**Affiliations:** 1 Division of Nephrology, Department of Medicine, St Michael’s Hospital, Toronto, ON M5B 1W8, Canada; Alejandro.Meraz-Munoz@unityhealth.to; 2 Consultant Nephrologist, Apollo Hospital, Navi Mumbai, Maharashtra 400614, India; drlangoteamit@gmail.com; 3 Division of Kidney Diseases and Hypertension, Donald and Barbara Zucker School of Medicine, Great Neck, NY 11021, USA; Kjhaveri@northwell.edu; 4 Department of Nephrology, Peupliers Private Hospital, Ramsay Générale de Santé, 75013 Paris, France; h.izzedine@ramsaygds.fr; 5 Division of Nephrology & Kidney Clinical Advancement, Research & Education Program, University of Cincinnati, Cincinnati, OH 45267, USA

**Keywords:** acute kidney injury (AKI), kidney replacement therapy (KRT), thrombotic microangiopathy (TMA), hematopoietic stem cell transplant (HSCT), tumor lysis syndrome (TLS)

## Abstract

Over the last three decades, advancements in the diagnosis, treatment, and supportive care of patients with cancer have significantly improved their overall survival. However, these advancements have also led to a higher rate of cancer-related complications. Acute kidney injury (AKI) and chronic kidney disease (CKD) are highly prevalent in patients with cancer, and they are associated with an increased risk of all-cause mortality. This bidirectional interplay between cancer and kidney, termed “the kidney–cancer connection” has become a very active area of research. This review aims to provide an overview of some of the most common causes of AKI in patients with cancer. Cancer therapy-associated AKI is beyond the scope of this review and will be discussed separately.

## 1. Introduction

**Figure 1:**
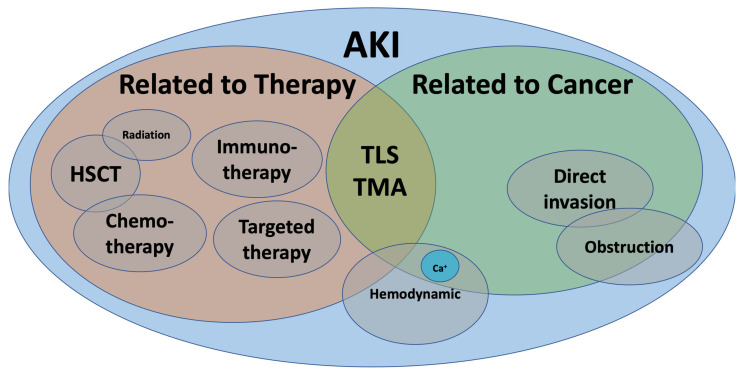
Acute kidney injury in cancer.AKI: acute kidney injury, Ca^+:^ calcium, HSCT: hematopoietic stem cell transplant, TLS: tumor lysis syndrome, TMA: thrombotic microangiopathy.

The last two decades have seen an exponential rise in the number of drugs used in cancer therapy. With precision medicine, novel targeted therapies, and better supportive care, the life expectancy of patients with cancer has improved [1]. Unfortunately, acute kidney injury (AKI) in the context of cancer has been increasingly recognized. Overall, AKI in cancer patients can be broadly classified as pre-renal or hemodynamic, intrinsic, and obstructive nephropathy. Nevertheless, malignancy adds a layer of complexity since AKI may be a direct complication of cancer itself (infiltration, paraneoplastic syndrome), cancer-related metabolic disturbances (hypercalcemia, tumor-lysis syndrome), anti-cancer therapy (chemotherapy, immune checkpoint inhibitors, stem-cell transplant) or other related complications (hypovolemia, infections, sepsis) (Figure 1).

## 2. Epidemiology of AKI in Patients with Cancer

Several different studies have defined the incidence of AKI related to cancer. Christiansen et al. described the incidence of AKI in all incident cancer patients in a population-based study in Denmark (*n* = 44, 116). The risk of developing AKI criteria was 17.5% during the first year and up to 27% during the first five years of cancer diagnosis [1]. A population-based study from Ontario, Canada, reported a cumulative incidence of AKI of 9.3% [2]. Similarly, a population-based study from China reported an incidence of AKI in patients with cancer of 7.5% [3]. The malignancies most frequently associated with AKI are multiple myeloma, kidney, liver, bladder and lymphoma and leukemia [4,5,6,7,8,9]. The risk factors for developing AKI are cancer stage, previous chronic kidney disease (CKD), diabetes mellitus and use of angiotensin-converting enzyme inhibitors (ACEi) or angiotensin receptor blockers (ARBs) [10]. Moreover, AKI in hospitalized patients has been linked to an increased length of hospital stay and costs of care [5,6].

## 3. Acute Kidney Injury in Critically Ill Patients with Cancer

Acute kidney injury occurs in 50–60% of patients admitted to the intensive care unit, and 20% of those patients have an underlying malignancy [7]. The frequency of AKI and kidney replacement therapy (KRT) has increased over the past couple of decades, perhaps due to better survival and a higher admission rate of patients with cancer to the intensive care units [1,8,9]. Critically ill patients with cancer are exceptionally susceptible to AKI, and the incidence of requiring KRT varies from 8–13% in patients with solid tumors and 10–34% in patients with hematological malignancies [10]. The risk of developing AKI is more significant in patients with septic shock, exposure to nephrotoxins, obstructive nephropathy and hematological malignancies, especially multiple myeloma [3,10,11,12] The short-term mortality of critically ill patients with cancer and severe AKI is comparable to critically ill patients without cancer [13,14]. In a cohort of critically ill patients with cancer (*n* = 975), the in-hospital and six-month mortality of AKI requiring KRT was 64% and 73%, respectively [12]. The need for KRT entails high mortality, and for patients who are not dialysis candidates, palliative care consult services can be helpful.

## 4. Hemodynamic Causes of Acute Kidney Injury in Patients with Cancer

Patients with cancer are susceptible to a myriad of hemodynamic insults. Oncology patients experience anorexia, nausea and vomiting in 60–80% of the cases [15]. A careful physical examination looking for signs of volume contraction should always be performed. Nonetheless, the sensitivity and specificity of physical examination are low. Initial investigations should include serum electrolytes, urea or blood urea nitrogen, and creatinine levels. A point of care ultrasound can further inform our evaluation in differentiating hypovolemia from other causes of AKI [16,17,18].

Hypercalcemia complicates up to 30% of all malignancies and causes AKI by several mechanisms [19]. Hypercalcemia leads to severe volume depletion via the activation of the calcium sensor located in the thick ascending loop of Henle, causing a furosemide-like effect [20]. Hypercalcemia leads to the vasoconstriction of the afferent arteriole, decreasing intra-glomerular pressure [21]. Finally, the precipitation of calcium phosphate crystals and clogging of the tubules have been described [22]. The initial treatment should be directed to restore intravascular volume with crystalloids (intravenous normal saline 200–250 mL/Hr). Loop diuretics are reserved for patients with volume overload. Anuric patients may become fluid overloaded rapidly and be unresponsive to diuretics; hence hemodialysis with low calcium baths should be performed in these cases. Calcitonin or bisphosphonates are indicated after the initial resuscitation [23]. The preferred bisphosphonates are pamidronate and ibandronate; however, zoledronic acid may be superior and has been used successfully in patients with serum Cr <4.5 mg/dL with a similar safety profile [24]. Zoledronic acid is not recommended for chronic use in patients with an estimated glomerular filtration rate of eGFR <30 mL/min/m2 [25]. Denosumab, a neutralizing monoclonal antibody directed against the receptor activator of nuclear factor kb ligand, has been used for the treatment of cancer-related hypercalcemia [26,27].

Contrast-associated acute kidney injury (CA-AKI) has been often cited as a common cause of AKI [28]. However, the relevance and causal relationship between contrast medium and intrinsic AKI have been questioned [29]. The use of iso-osmolar and low-osmolar contrast agents in low quantities has improved these procedures’ safety [30]. The rise in serum creatinine associated with contrast may be explained by intraglomerular hemodynamic changes rather than intrinsic tubular damage [31,32]. In patients with an estimated glomerular filtration rate (eGFR) of >45 mL/min/1.73 m^2^, the risk of AKI is negligible. Caution is advised in patients with a lower eGFR who also have other risk factors for CA-AKI. For these patients, prophylactic IV fluids are advised [33].

Heart failure (HF) is another hemodynamic derangement commonly encountered in patients with cancer. Besides the common causes of HF, it is essential to inquire about previous exposure to cardiotoxic chemotherapy such as anthracyclines (doxorubicin, daunorubicin, and epirubicin) and the human epidermal growth factor receptor 2 (HER2) modulator trastuzumab [34]. Type 1 cardiorenal syndrome is the result of a decreased eGFR secondary to kidney hypoperfusion. Low cardiac output and intra-renal venous congestion are the main drivers of this pathological condition. Maladaptive neurohormonal changes such as the upregulation of the renin–angiotensin–aldosterone system (RAAS), the non-osmotic release of vasopressin and the sympathetic nervous system’s activation result in increased sodium and water reabsorption [35,36]. Decongestion with a loop diuretic alone or in combination with other classes of diuretics are the first step in treating this pathology [37].

Liver injury or cirrhosis are associated with hepatorenal syndrome (HRS), a diagnosis of exclusion. Portal hypertension causes nitric oxide-mediated splanchnic vasodilation, with secondary pooling of blood in the splanchnic circulation and hypotension. Similarly, the activation of RAAS and other neurohumoral systems leads to kidney vasoconstriction, hypoperfusion, and the retention of salt and water. Management includes treating HRS precipitants and restoring effective arterial circulation with vasopressors: terlipressin, norepinephrine or midodrine in combination with octreotide [38].

Tense ascites may increase intraabdominal pressure, causing abdominal compartmental syndrome. Paracentesis with albumin replacement may improve kidney hemodynamics and help alleviate AKI [39]. The sinusoidal obstruction syndrome, a complication of stem-cell transplant, is similar to hepatorenal syndrome due to the associated portal hypertension secondary to the hepatic sinusoidal injury. 

## 5. Cancer-Associated Thrombotic Microangiopathy (TMA)

**Figure 2:**
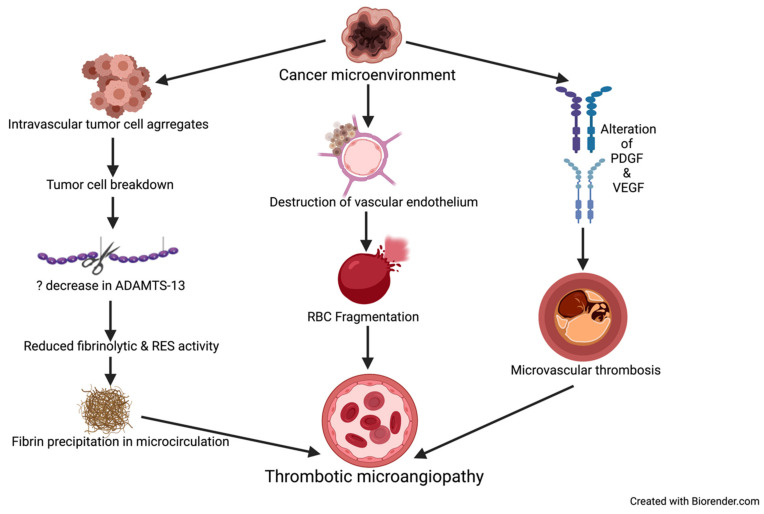
Mechanisms causing TMA in patients with cancer. ADAMTS-13 = a disintegrin and metalloproteinase with thrombospondin type-1 motif, member 13, PDGF = platelet derived growth factor. VEGF = vascular endothelial growth factor, RBC = red blood cell, RES = reticuloendothelial system.

Cancer-associated thrombotic microangiopathy (TMA) refers to a constellation of disorders characterized by microvascular thrombosis, thrombocytopenia, and resultant ischemia of the end organ affected, e.g., kidney and brain [40]. The pathological characteristics of TMA include intrarenal or systemic microvascular thrombosis with endothelial swelling and microvascular obstruction (Figure 2) [41].

TMA syndromes are a complication of cancer itself and can also occur as a side effect of cancer chemotherapeutic agents [42]. One of the earliest reported studies on TMA in cancer patients is from 1972 from Germany, which showed 5.7% of patients with metastatic cancer have TMA [43]. Gastric carcinoma tops the list (50%), followed by breast and lung carcinoma (Table 1).

### 5.1. Differentiating Cancer-Associated TMA and Cancer Chemotherapy-Induced TMA

In oncological practice, one must differentiate between cancer-associated and chemotherapy-induced TMA. In some cases, it is very challenging to delineate the two. Table 2 outlines some key differences between the two processes. 

### 5.2. Treatment of Cancer-Associated TMA

In general, plasma exchange is more effective than plasma infusion in treating thrombotic thrombocytopenic purpura (TTP) because its pathogenesis is mediated by ultra-large Von Willebrand Factor (VWF) multimers and autoantibodies to a disintegrin and metalloproteinase with thrombospondin type-1 motif, member 13 (ADAMTS-13). These mediators are at least partially removed by plasma exchange [54]. Cancer-associated TMA responds poorly to plasma exchange, most likely due to the lack of severe deficiency of ADAMTS-13 [55]. Cancer-associated TMA has been observed to improve with the control of the underlying cancer [56].

## 6. Acute Kidney Injury Due to Renal Parenchymal Invasion/Infiltrative Malignancies

Many solid and hematological cancers may involve the renal parenchyma. Lymphomas and leukemias are the most common cancers that demonstrate autopsy evidence of infiltration, with the incidence being 6% to 60% [57,58]. Lymphomatous invasion of the kidneys (LIK) can present as acute kidney injury, new-onset or worsening proteinuria and hematuria; however, diagnosis is usually incidental. In approximately one percent of cases, the tumor burden infiltrating the kidneys can be so high that it can lead to AKI [59,60]. Tornroth et al. demonstrated various pathological phenotypes of lymphomatous invasion of kidneys [61]. Most cases (87%) showed interstitial infiltration followed by intraglomerular infiltration (45%). Renal imaging in these cases shows bulky and enlarged kidneys. A high index of suspicion is necessary to prompt a kidney biopsy. Another case series showed that 34% of non-Hodgkin’s lymphoma developed parenchymal kidney invasion; however, only 14% were diagnosed before death [62]. For indolent hematological cancers such as chronic lymphocytic leukemia, which are often not treated unless there is end-organ involvement, the demonstration of LIK may often pull the trigger to initiate chemotherapy. The most common solid organ cancers metastasizing to the kidneys are lung carcinoma, gastric, breast and malignant melanoma [11]. Renal metastases usually manifest as bilateral, small, multifocal parenchymal nodules, though single exophytic lesions have also been described [63]. Metastases to kidneys are seen in a setting of massive tumor burden and portend a poor prognosis. Acute kidney injury from infiltrative cancers results from renal parenchymal compression, which leads to the disruption of the glomerular, tubulointerstitial and microvascular architecture, leading to impairment of the GFR. Most cases are subclinical; however, patients may present with hypertension (the upregulation of the renin–angiotensin axis), flank pain (due to stretching of renal capsule) and hematuria.

## 7. Hematopoietic Stem Cell Transplant-Related Acute Kidney Injury

Acute kidney injury after hematopoietic stem cell transplant (HSCT) is usually defined as a doubling of baseline serum creatinine or decline in GFR of at least 50% within the first 100 days after engraftment [64]. It was difficult to determine the epidemiology of HSCT-related AKI due to inconsistencies in the AKI definition. Hence, an attempt was made to develop uniformity to gain insight into the epidemiology of AKI and facilitate the comparison of studies. Recent studies have used criteria such as the risk, injury, failure, loss of kidney function, end-stage kidney disease (RIFLE) system and the Acute Kidney Injury Network (AKIN) criteria for kidney injury. A doubling of the serum creatinine level is correlated with RIFLE-I (injury to the kidney) and AKIN stage 2 [64].

AKI occurs in 12–21% of patients undergoing autologous HSCT and majorly depends on the type of conditioning used after allogenic HSCT. Myeloablative conditioning and reduced-intensity conditioning (RIC) are associated with 35–56% and 7–46% incidence of AKI [64,65,66,67,68,69]. Kidney replacement therapy requirement in autologous HSCT, myeloablative allogenic HSCT and RIC is 7%, 20–33% and 4%, respectively [70]. Not only AKI occurring within the first 30 days of HSCT, but also its severity portends an an increased risk of death and overall low survival rates [64,71,72]. Among patients who require KRT, the mortality is exceedingly high (55–100%) [73,74].

### 7.1. Pathogenesis of HSCT-Related AKI

**Figure 3:**
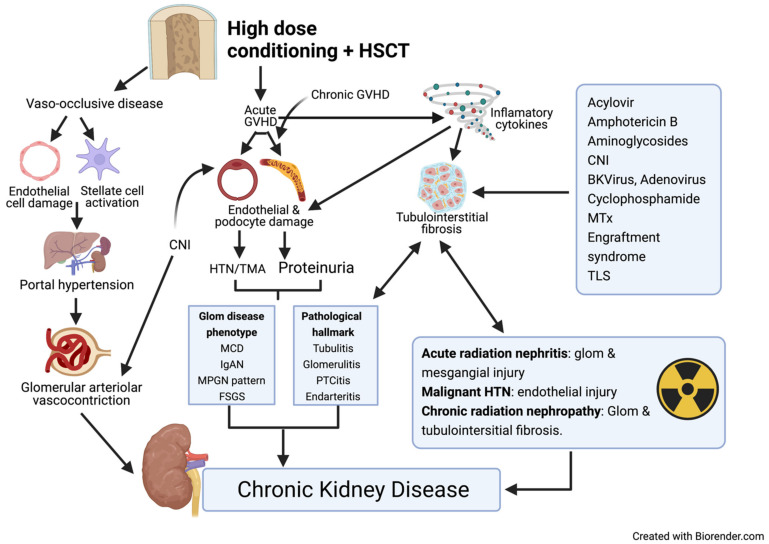
Pathogenesis of HSCT-associated AKI. HSCT = hematopoietic stem cell transplant, GVHD = graft vs. host disease, Glom = glomerular, MCD = minimal change disease, IgAN = IgA nephropathy, MPGN = membranoproliferative glomerulonephritis, FSGS = focal segmental glomerulosclerosis, PTCitis = peritubular capillaritis, CNI = calcineurin inhibitor, MTx = methotrexate, TLS = tumor lysis syndrome, HTN = Hypertension.

Hematopoietic stem cell transplantation can induce a myriad of pathophysiological changes in virtually all compartments of renal parenchyma (Figure 3).

### 7.2. Risk Factors and Etiology of HSCT-Related AKI

Traditional cardiovascular risk factors such as diabetes mellitus or hypertension predispose patients to develop AKI post-HSCT. However, there are transplant-specific factors in both myeloablative and non-myeloablative groups. These risk factors are along with various etiologies and their pathological phenotypes are outlined in Table 3.

## 8. Tumor Lysis Syndrome

Tumor lysis syndrome (TLS) is one of the most common oncological emergencies encountered in clinical practice. 

### 8.1. Definition of TLS

Tumor lysis syndrome was defined by Hande-Garrow in 1993 and by Cairo-Bishop in 2004 [77,78], later modified by Howard and colleagues in 2011 [79]. Cairo-Bishop’s definition (Table 4) proposed specific laboratory criteria and grading for TLS [80,81].

### 8.2. Epidemiology

The incidence of TLS is not well defined due to a lack of a universal definition, heterogeneous patient population, and differences in treatment strategies and prophylaxis. Bulky and rapidly proliferating tumors, as well as treatment-sensitive tumors are at high risk of TLS. Incidences of TLS, ranging from 4% to 53%, have been quoted in childhood hematological malignancies with acute lymphoblastic leukemia (ALL), Burkitt and diffuse large B-cell lymphoma carrying the highest risk [82]. Pre-treatment kidney failure (Sr creatinine >1.4 mg/dL) strongly predicted TLS and clinical TLS was associated with higher mortality than lab TLS [83]. However, with the advent of newer and effective targeted therapies such as monoclonal antibodies, immunotherapy and drugs such as tyrosine kinase inhibitors, TLS has been reported in these low-risk tumors as well.

### 8.3. Risk Factors and Risk Assessment

Risk factors for developing TLS can be broadly classified into disease-related, patient-related and treatment related factors (Table 5).

### 8.4. Prevention and Treatment of Tumor Lysis Syndrome (TLS)

**Figure 4:**
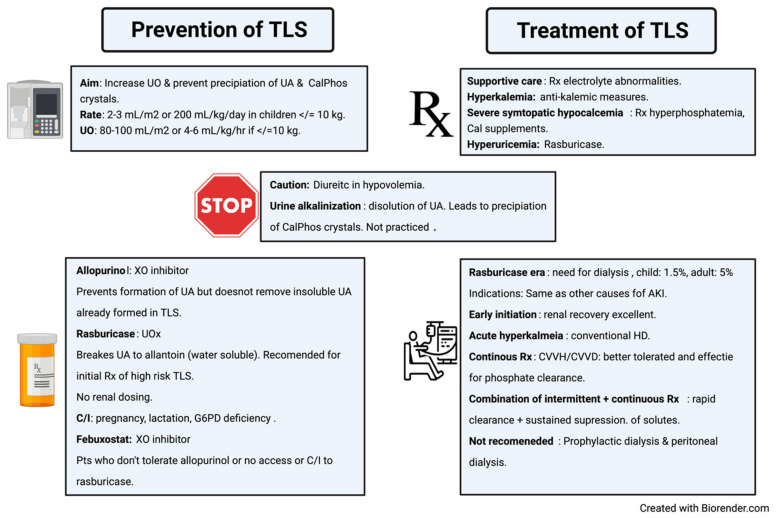
Infographic that summarizes prevention and treatment of tumor lysis syndrome. TLS = tumor lysis syndrome, UO = urine output, CalPhos = calcium phosphate, UA = uric acid, XO = xanthine oxidase, Rx = treatment, C/I = contraindication, G6PD = glucose 6 phosphate dehydrogenase, AKI = acute kidney injury, HD = hemodialysis, CVVH/HD = continuous veno venous hemofiltration/hemodialysis.

Tumor lysis syndrome can have dire complications; hence the International Panel on TLS recommends preventive and treatment strategies [80]. These strategies have been outlined in the infographic (Figure 4).

## 9. Obstructive Uropathy

Malignancies of the genitourinary tract commonly predispose to urinary tract obstruction. These commonly include cancer of the bladder, prostate, uterus and cervix. Obstruction typically occurs due to intratubular blockage or extrarenal obstruction by a tumor mass. Intratubular obstruction is commonly seen in lymphoproliferative malignancies and occurs due to crystals precipitating in tubules, e.g., uric acid, light chain casts, drug crystals, e.g., high dose methotrexate therapy, blood clots, among others. Extrarenal obstruction commonly occurs in metastatic disease of gastrointestinal and genitourinary tracts, e.g., retroperitoneal tumor or fibrosis or enlarged lymph nodes, bladder obstruction due to cancer, urothelial cancer of ureter, post-radiation therapy (immediately due to blood clots or later due to fibrosis involving the ureteral orifices), BK virus infection in HSCT patients, etc.

Non-dilated obstructive uropathy is frequently missed as causative of AKI, and it is an underreported condition [84]. It is commonly seen in malignancies of pelvic areas where there is minimal or no dilatation of the proximal part of the urinary tract; however, percutaneous nephrostomy leads to rapid renal recovery. The possible causes for the non-dilatation of ureters are the encasement of the ureter with a tumor or fibrous tissue [85], abnormal ureteral peristalsis [86], ureteral edema [87,88] or simultaneous severe volume depletion. Hence, in unexplained renal failure in pelvic malignancies, obstructive uropathy must be ruled out even in the absence of pelvicalyceal system (PC)system dilatation on imaging.

Clinically, patients may be asymptomatic despite chronic urinary tract obstruction. Acute obstruction may lead to pain and hematuria. Urine output may persist unless complete bilateral ureteric obstruction occurs. Hence, the presence of urine does not rule out obstruction.

Imaging may show dilated ureter and collecting systems in one or both kidneys. Ultrasound is commonly carried out as the first imaging modality due to its easy availability and no radiation exposure. Alternatively, CT scan, MRI or nuclear imaging can also be used. Antegrade urography can establish the level of obstruction in patients who have nondiagnostic imaging studies.

Once diagnosed, percutaneous nephrostomy (PCN) or ureteral stents are used to relieve the obstruction. In a retrospective study of 102 patients who underwent decompression for ureteral obstruction, 68% had a bilateral obstruction. Stent or PCN placement was successful in 95% of cases, but more than 50% of patients developed complications such as urinary tract infection. The presence of metastasis and malignant ureteral obstruction in previously established malignancy were independent prognostic factors for inferior overall survival [89]. The median survival was less than seven months in such patients. Clinical judgement is required to decide which patients would benefit most from decompression with these invasive procedures [90]. A multidisciplinary decision-making process is highly recommended in cases with advanced malignancy.

## 10. Conclusions

Acute kidney injury is a global health problem and patients with cancer are more susceptible to it. The occurrence of AKI is a predictor of all-cause mortality in patients with cancer. Dramatic advances have occurred in management which has prolonged the longevity of life in patients with cancer. However, this comes at an expense, with an increase in the drug- and cancer-related adverse renal events. AKI can be due to cancer itself or as a complication of its treatment and poses a challenge to the health care personnel. The etiology is becoming increasingly complex and multifactorial, and a physician needs to keep an open-minded approach to treat these patients in an optimal manner. A systematic evaluation of the patient is essential to identify potential causes for AKI as it can have treatment and prognostic implications. Kidney biopsy must be considered whenever deemed safe, in unexplained AKI and in cases where immune-mediated renal injury is suspected.

The emergence of onco-nephrology as a subspecialty falls at an intersection of oncology and nephrology care. A multidisciplinary team is the need of the hour and should include an oncologist, nephrologist, dedicated nursing team, nutritionist, palliative care and primary care physician. The focus needs to be shifted to manage patients’ comorbidities such as diabetes, hypertension, dyslipidemia and cardiovascular disease, as these are known to predispose patients with cancer to AKI. The prevention of AKI is of paramount importance to prevent the downstream effects on the patient’s health. In the current era of precision medicine, we are still in search of a perfect biomarker which could potentially predict AKI occurrence before the renal dysfunction sets in. With our increasing understanding of the pathophysiology of AKI in this niche population, hopefully the outcomes of patients suffering with cancer will improve and mitigate kidney-related adverse events.

## Figures and Tables

**Table 1:** Cancers associated with TMA [44,45,46,47,48,49].

System.	Cancers.
**Gastrointestinal Tract**	Gastric Cancer Colon Cancer Carcinoma of the Anal Canal (Squamous Cell Carcinoma) Metastatic Appendiceal Carcinoma
**Lung**	Adenocarcinoma Squamous Cell Carcinoma Small Cell Lung Cancer
**Genitourinary Tract**	Prostate Cancer Ovarian Cancer Renal Cell Carcinoma Seminal Vesicle Tumor.
**Hepatobiliary System**	Hepatocellular Carcinoma Pancreatic Cancer Cholangiocarcinoma
**Endocrine System**	Multiple Endocrine Neoplasia Type 1 Pheochromocytoma Neuroendocrine Tumor Prolactin-Producing Pituitary Adenoma
**Hematologic Malignancies**	Non-Hodgkin Lymphoma Acute Lymphoblastic Leukemia Myelodysplastic Syndrome Hodgkin Lymphoma Multiple Myeloma
**Others**	Breast Cancer Kaposi Sarcoma Carcinoma of Unknown Origin

**Table 2:** Key differences between cancer-related and chemotherapy-related TMA [46,50,51,52,53]. TTP: thrombotic thrombocytopenic purpura.

Features	Cancer-Associated TMA	Chemotherapy-Induced TMA
**Metastatic Disease**	90% Have Metastatic Disease	May not be Present.
**Clinical Phenotype**	TTP-Like Phenotype in Approximately 6% of Patients	The Spectrum Ranges from Typical HUS to Kidney Limited TMA
**Disseminated Intravascular Coagulation**	Present in 15% of Patients	Absent
**Blood Picture**	Carcinocythemia (Carcinoma Cell Leukemia) is Associated with TMA Seen on Peripheral Blood Film in Disseminated Solid Organ Malignancies Leucoerythroblastic with More Significantly Elevated Serum LDH Levels	These Phenomena May or May not Occur
**Mean Age**	56 Years	40 Years
**Symptoms**	Progressive Weakness, Weight Loss and Pain. The Median Duration of Symptoms is 21 Days	The Median Duration of Symptoms is 8 Days
**Therapy**	Fail to Respond to Plasmapheresis	Those with ADAMTS13 Deficiency Especially Respond to Plasmapheresis

HUS = Hemolytic Uremic Syndrome, LDH = Lactate Dehydrogenase.

**Table 3:** Risk factors, etiology and pathological phenotype of AKI in HSCT [70,75,76].

Risk Factors for AKI Post HSCT	Etiologies of AKI	Pathology
**Common Risk Factors** Pre-HSCT DM Pre-HSCT HTN Pre-HSCT Renal Impairment Sepsis Amphotericin Mechanical Ventilation ICU Admission **Myeloablative HSCT** Hepatic SOS Lung Toxicity High-Risk Disease Acute GVHD CNI **Non-Myeloablative HSCT** Prior Myeloablative HSCT CMV Reactivation High-Risk Disease Acute GVHD CNI MTx	**Prerenal** Dehydration ES Sepsis Hepatic SOS	ATN Acute HRS Physiology
	**Glomerular**	TA-TMA, MCD, MN, MPGN & FSGS Pattern
	**Ischemic Injury** Dehydration ES Sepsis Shock	ATN ATN ATN ATN
	**Direct Nephrotoxicity** Drugs (Chemotherapy, Antimicrobials, CNI, MTx) Marrow Transfusion Toxicity Acute GVHD BK Virus & Adenovirus	Tubulointerstitial Damage ATN, Intratubular Obstruction due to Hemolysis from DMSO. AIN AIN
	**Tumor Lysis Syndrome**	Intratubular Obstruction ATN
	**Postrenal Obstruction** Retroperitoneal Fibrosis Lymphadenopathy Hemorrhagic Cystitis BK Cystitis Adenovirus Cystitis	

DM = diabetes mellitus, HSCT = hematopoetic stem cell transplant, HTN = hypertension, SOS = sinusoidal obstruction syndrome, GVHD = graft vs host disease, CNI = calcineurin inhibitor, CMV = cytomegalovirus, MTx = methotrexate, ES = engraftment syndrome, HRS = hepatorenal syndrome, TA-TMA = transplant associated thrombotic microangiopathy, MCD = minimal change disease, MPGN = membranoproliferative glomerulonephritis, MN = membranous nephropathy, FSGS = focal segmental glomerulosclerosis, ATN = acute tubular necrosis, DMSO = dimethyl sulfoxide.

**Table 4:** Cairo-Bishop’s definition for laboratory and clinical tumor lysis syndrome (TLS).

Laboratory TLS				Clinical TLS		
Two or More Laboratory Abnormalities from Day 3 up to Day 7, After Initiation of Cytotoxic Therapy: Uric Acid ≥8 mg/dL Potassium ≥6 mEq/L Phosphate ≥6.5 mg/dl for Children or ≥4.5 mg/dl for Adults Calcium ≤7mg/dL OR 25% Change from Baseline in Any of the Above Values				Laboratory TLS Plus 1 or More of the Following: Creatinine ≥1.5 times the upper limit of normal (ULN) Cardiac Arrhythmia Seizure Sudden Death		
**Complication**	**Cairo Bishop Grading of Clinical Tumor Lysis Syndrome**					
	**Grade**					
	**0**	**1**	**2**	**3**	**4**	**5**
**Laboratory TLS**	Absent	Present	Present	Present	Present	Present
**Creatinine**	<1.5 Times ULN	1.5 Times ULN	1.5 to 3.0 Times ULN	>3.0 to 6.0 Times ULN	>6.0 Times ULN	Death
**Cardiac Arrhythmia**	None	Intervention not Indicated	Nonurgent Medical Intervention Indicated	Symptomatic Despite Medications, Controlled with a Device (e.g., Defibrillator)	Life-Threatening and Associated with congestive heart failure Syncope, Shock	Death
**Seizures**	None	Not applicable	One Brief Generalized Seizure; Seizure(s) Well controlled by Anticonvulsants; Infrequent Focal Motor Seizures not Interfering with Activities of Daily Living	Seizure with Altered Consciousness; Poorly Controlled Seizure Disorder with Breakthrough Generalized Seizures Despite Medical Management	Intractable Seizure, Status Epilepticus	Death

**Table 5:** Risk Factors for Tumor Lysis Syndrome.

Risk Factors for Tumor Lysis Syndrome		
Disease-related	Patient-related	Treatment-related
Rapid Cellular Proliferation (LDH >2-Time ULN) High Tumor Burden (Tumor >10 cm, metastatic disease, WBC >25 × 10^3^/μL) Sensitive to Cytoreductive Therapy Renal Infiltration or Outflow Tract Obstruction	Preexisting Renal Disease Preexisting Hyperuricemia Hypovolemia Hypotension Acidic Urine	Intensity of Cytoreductive Therapy (Single Agent Versus Combination, Disease Specific) Concomitant Use of Nephrotoxic Drugs Inadequate Hydration During Treatment
